# Supplementary material for: Experimental study on factors influencing low identification rates and spectral quality of Streptococcus pneumoniae using the Sepsityper kit
Source: Microbiol Spectr. 2025 Jul 31;13(9):e01084-25. doi: 10.1128/spectrum.01084-25 (PMC12403568; doi:10.1128/spectrum.01084-25)
Supplement: Supplemental Material — Tables S1 to S3; Fig. S1. [file spectrum.01084-25-s0001.docx]

**Supplemental materials**

**Table S1.** The characteristics of *S. pneumoniae* ATCC 49619 and five clinical strains of *S. pneumoniae*

| Strain | Origin | Serotype |
| --- | --- | --- |
| ATCC 49619 |  | 19A |
| P01 | Blood | 35B |
| P02 | Blood | 10A |
| P03 | Blood | 3 |
| P04 | Blood | 35B |
| P05 | Blood | 15C |

**Table S2.** Pilot study results of factors influencing correct species-level identification rates of *S. pneumoniae* ATCC 49619

| Volume of lysis buffer | Sample preparation method | MALDI-TOF MS platform | MALDI Biotyper sirius | VITEK MS | MALDI Biotyper sirius | VITEK MS | MALDI Biotyper sirius | VITEK MS | MALDI Biotyper sirius | VITEK MS |
| --- | --- | --- | --- | --- | --- | --- | --- | --- | --- | --- |
|  |  | Blood culture system | BD BACTEC FX | | BACT/ALERT VIRTUO | | BD BACTEC FX | | BACT/ALERT VIRTUO | |
|  |  | Bottle type | Aerobic bottle | | | | Anaerobic bottle | | | |
| 200 μL (standard method) | DT |  | 0/9 | 0/9 | 0/9 | 0/9 | 0/9 | 2/9 | 0/9 | 0/9 |
|  | eDT |  | 0/9 | 0/9 | 0/9 | 0/9 | 0/9 | 0/9 | 0/9 | 1/9 |
|  | Ext |  | 0/9 | 0/9 | 0/9 | 0/9 | 1/9 | 0/9 | 2/9 | 1/9 |
| 100 μL (Cordovana method) | DT |  | 0/9 | 0/9 | 0/9 | 0/9 | 0/9 | 0/9 | 1/9 | 0/9 |
|  | eDT |  | 0/9 | 0/9 | 0/9 | 0/9 | 0/9 | 0/9 | 1/9 | 0/9 |
|  | Ext |  | 0/9 | 0/9 | 0/9 | 0/9 | 0/9 | 0/9 | 0/9 | 3/9 |

DT, direct transfer; eDT, extended direct transfer; Ext, full extraction.

**Table S3.** Validation study results of factors influencing correct species-level identification rates of five clinical strains of *S. pneumoniae.*

| Lysis buffer volume | Sample preparation method | Blood culture system | BD BACTEC FX | BACT/ALERT VIRTUO | BD BACTEC FX | BACT/ALERT VIRTUO |
| --- | --- | --- | --- | --- | --- | --- |
|  |  | Bottle type | Aerobic bottle | | Anaerobic bottle^a^ | |
| 200 μL  (Standard method) | DT |  | 0/10 | 0/10 | 0/4 | 0/8 |
|  | eDT |  | 0/10 | 0/10 | 0/4 | 0/8 |
|  | Ext |  | 2/10 | 0/10 | 0/4 | 2/8 |
| 100 μL  (Cordovana method) | DT |  | 1/10 | 0/10 | 0/4 | 3/8 |
|  | eDT |  | 1/10 | 0/10 | 0/4 | 2/8 |
|  | Ext |  | 0/10 | 0/10 | 0/4 | 4/8 |

DT, direct transfer; eDT, extended direct transfer; Ext, full extraction.

MALDI-TOF MS analysis was performed exclusively in single or duplicate using the MALDI Biotyper sirius.

^a^Due to a delay in the positive alarm, one BACT/ALERT anaerobic bottle and three BD BACTEC Plus Aerobic/F bottles were excluded from the study.


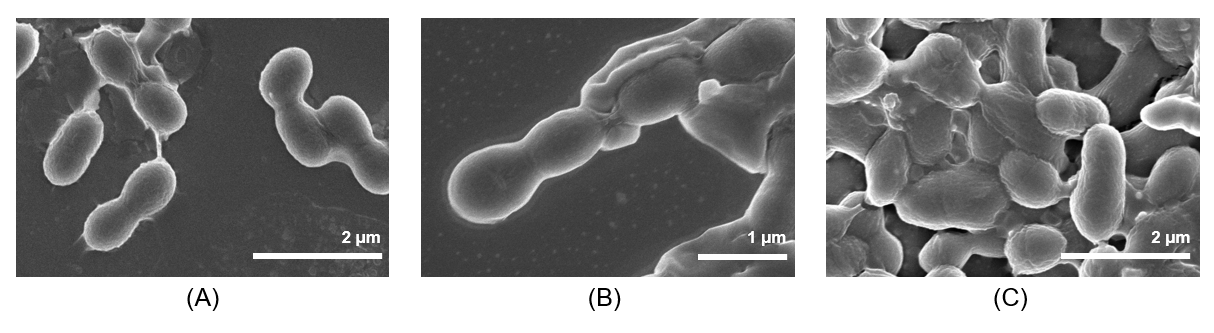


**Figure S1.** Scanning electron microscopy (SEM) images of *S*. *pneumoniae* ATCC 49619 under different conditions. (A) Cells collected from a BACT/ALERT aerobic bottle containing 10 mL of whole blood after a positive alarm. (B) Cells collected from a BACT/ALERT aerobic bottle containing 10 mL of PBS after a positive alarm. (C) Control cells collected after 18 h of culture on blood agar plates (BAP) at 37°C with 5% CO_2_.
